# Moving Toward Participation‐Focused Goals for Students With Disabilities: Outcomes of a Knowledge Translation Program for School‐Based Therapists

**DOI:** 10.1111/cch.70313

**Published:** 2026-07-08

**Authors:** Michal Waisman Nitzan, Yonat Ivzori, Barkin Kose, Dana Anaby

**Affiliations:** ^1^ Department of Occupational Therapy University of Haifa Haifa Israel; ^2^ Yonat Ivzori, Department of Occupational Therapy Zefat Academy College Zefat Israel; ^3^ Department of Special Education Oranim Academy College Kiryat Tiv'on Israel; ^4^ Department of Occupational Therapy, Faculty of Gülhane Health Sciences Health Sciences University Ankara Turkey; ^5^ School of Physical and Occupational Therapy McGill University Montreal Quebec Canada

**Keywords:** goal formulation, knowledge‐translation, participation, school‐based practice

## Abstract

**Background:**

Participation in meaningful school activities is a key outcome of interventions in allied health. Although participation‐focused approaches exist, their integration into school‐based practice remains limited. Goal formulation is central to individualized education plans (IEPs), which often emphasize skills or impairments rather than participation in activities. This study aimed to examine changes in goals set by school‐based therapists and students with disabilities following a participation‐focused knowledge translation (KT) programme.

**Methods:**

School‐based therapists (*n* = 101) completed a 30‐h, eight‐session, 15‐week training on participation. In the first session (T1), therapists provided an example of one or more IEP goals for one student on their caseload (*n* = 135). In the fifth session (T2), therapists set a new goal for the same student (*n* = 95). Generalized Linear Mixed Models (GLMMs) with therapist as a random intercept were used to compare goals against seven participation‐focused criteria across the two time points. Goals were also categorized into participation domains and descriptively compared across time points.

**Results:**

Significant changes in goal formulation were observed across all seven criteria (*p* < 0.001) from T1 to T2: The estimated probability of a goal being activity‐focused increased from 56% to 96%, situated in real‐life settings from 55% to 95% and addressing attendance from 17% to 74%, involvement from 14% to 45%, social context from 54% to 91% and opportunity to choose from 46% to 84%. The probability of a goal targeting skills or body functions decreased from 38% to 5%. Although formal education remained a dominant domain, more goals addressed social participation (76.8% vs. 32.8%), play (41.0% vs. 20.3%) and leisure (24.2% vs. 7.4%).

**Conclusions:**

Goals formulated during the KT programme expanded beyond academics to include play, leisure and social participation. Further implementation studies are needed to ensure the change is sustained beyond the training period.

## Summary

- Participation‐focused KT programmes hold promise in supporting a change in goal‐formulation, moving from impairment‐ and skill‐based objectives toward more contextually grounded, participation‐focused goals.
- School‐based health professionals may benefit from considering play, leisure and social participation as well as academic goals because these areas contribute to children's well‐being and holistic development.
- The participation‐focused goal criteria may serve as a practical checklist to guide therapists in evaluating whether goals reflect essential aspects of participation.

## 1 Introduction

*Participation*, as defined by the International Classification of Functioning, Disability and Health, refers to involvement in life situations (World Health Organization [WHO] 2001). It is central to students' development and well‐being in school (Leonardi et al. 2022). The family of participation‐related constructs further conceptualizes participation as comprising two dimensions: attendance (being there) and involvement (the experience while attending). This framework provides conceptual clarity to guide research and practice in childhood disability by illustrating how participation relates to activity competence, sense of self, preferences and the environment/context (Imms et al. 2017). Opportunities and choices, shaped by personal and environmental factors, can support the translation of school and therapeutic activities into students' real‐life participation (Cogan and Carlson 2018; Maciver et al. 2023). Consistent participation in meaningful school and peer activities further supports skill development, identity formation and a sense of belonging, with long‐term benefits for mental health and quality of life (Homman et al. 2025; Kaelin et al. 2024). The multidimensional conception of participation is also reflected in the UN Convention on the Rights of Persons with Disabilities (United Nations 2006), which affirms participation in all areas of daily life as a fundamental right for people with disabilities. Together, these frameworks represent a multidimensional, biopsychosocial conceptualization of participation that integrates individual, relational and environmental dimensions.

However, despite its importance, school participation remains restricted for many students with disabilities. Individual‐ environmental‐ and organizational‐level barriers limit their involvement in classroom tasks, peer activities and social/recreational life (Bonnard et al. 2022; Coster et al. 2013; Maciver et al. 2019, 2023). To illustrate, parents frequently report that their children with disabilities rarely join clubs or school‐sponsored teams because they lack the necessary support (Coster et al. 2013; Mei et al. 2015). Such restrictions can reduce academic engagement, weaken peer relationships and hinder transition to adulthood roles (Kaelin et al. 2021; Simeonsson et al. 2001).

Knowledge‐to‐practice gaps contribute to these inequities. Although participation‐focused assessments and interventions are well‐documented (Anaby et al. 2021), their routine integration in schools is uneven. Teachers and other practitioners may face challenges in effectively implementing participation‐focused strategies due to gaps in their training and limited conceptual clarity around participation (Maciver et al. 2023). The school environment also shapes a student's feelings of belonging and involvement. Factors like accommodations, teacher–student relationships and the social atmosphere among peers can limit or support participation (Kara et al. 2025; Maciver et al. 2019).

Practice audits indicate that routine services infrequently prioritize participation. A UK audit using the Method for using Audit and Feedback in Participation Implementation showed participation outcomes specified as goals in approximately 30% of cases, child and parent involvement in approximately 13% and measured progress in approximately 20% (Kolehmainen et al. 2020). Consistent with these findings, a New Zealand study reported that goal‐setting was not routinely implemented and barriers limited family and interprofessional involvement, particularly during participation‐focused goal‐setting (Graham et al. 2020). Overall, routine paediatric rehabilitation has insufficiently prioritized participation outcomes, inadequately involved stakeholders and addressed the school context only to a limited extent (Teleman et al. 2021).

Although goal formulation is essential for bridging the knowledge‐to‐practice gap, it remains underused. Current practices frequently fail to leverage participation‐focused goals that connect educational supports to student‐valued outcomes. Instead, professionals tend to set goals centred on addressing impairments or specific tasks, without sufficiently considering the environmental factors essential for authentic participation (Rens and Joosten 2013). Related goal‐setting research has demonstrated a significant disconnect between recommended practice and reality. Goals are frequently written in technical language that does not capture the child's lived experience or the family's perspective, and in most cases, they do not explicitly target participation (Kolehmainen et al. 2020; Ryan et al. 2025).

Implementing knowledge translation (KT) is necessary to move participation research into everyday school practice and improve how goals are set (Anaby et al. 2021). However, few programs apply KT frameworks to implement occupation‐based, participation‐focused, context‐responsive approaches. Ray et al. (2024) KT study showed that a training program for school‐based occupational therapists led to shifts toward participation‐focused interventions, increased in‐context service delivery and stronger interdisciplinary collaboration. Their study highlighted additional impacts, including enhanced practitioner confidence, improved performance‐based goal writing and deeper integration within school teams.

The pathways and resources for engagement and participation (PREP) framework provides a systematic approach to achieving and enhancing participation by addressing environmental barriers, utilizing existing resources and fostering meaningful participation outcomes within a student's natural environment (Law et al. 2016). A KT study on the PREP approach, reported in two complementary papers, provides compelling practice‐based evidence. The study involved therapists and managers, who demonstrated strong PREP knowledge through a validated vignette after a tailored KT intervention in inclusive schools across Israel. They reported a shift in their practice toward environmental strategies and broader partnerships, such as with classmates and community instructors. This reorientation led to the adoption of participation goals meaningful to the child. At the student level, activity performance and satisfaction (assessed using the Canadian Occupational Performance Measure) improved from baseline to postintervention and follow‐up (Waisman‐Nitzan et al. 2022, 2023). Our study aims to examine changes in the scope and focus of goals set by school‐based therapists and students with disabilities during their participation in a PREP‐based KT intervention.

## 2 Methods

### 2.1 Design

This KT study strategy involved a series of four participation‐focused training programs offered by the Israeli Ministry of Education between 2020 and 2023. The programs were grounded in the Participation‐KT roadmap (Anaby et al. 2021), a framework specifically developed to guide the translation of participation‐focused evidence into practice. This framework provided the conceptual basis for the design, delivery and evaluation of the intervention. The programs, each consisting of eight sessions delivered over 15 weeks, targeted school‐based therapists working with children with disabilities in various educational settings. They included didactic and interactive sessions, structured assignments between sessions and ongoing coaching from the research team. The curriculum encompassed theories about participation, environmental analysis, participation‐focused measures, strategies to improve participation and case studies. The program focused on utilizing PREP (Law et al. 2016) in real‐life school settings. A detailed description of the program content, teaching methods and implementation components has been previously published (Waisman‐Nitzan et al. 2022). Goal formulation was evaluated at two points during the program. Informed consent was obtained from all participants, and parents provided consent for the use of their child's de‐identified data. The study was approved by the Ethics Committee of the Israeli Ministry of Education and the Institutional Review Board of McGill University.

### 2.2 Procedure

One hundred and one school‐based therapists from across Israel participated in the KT programs as part of a professional development course offered by the Ministry of Education. The programs aimed to expand participants' knowledge and perceptions of participation‐focused, evidence‐based practice and to support the translation of this knowledge into goal‐setting and intervention practices (Waisman‐Nitzan et al. 2022, 2023).

At T1 (first session), therapists provided one or more example IEP (Individualized Education Program) goals for one student from their caseload (*n* = 135). At T2 (fifth session), they set a new goal for the same student to be implemented in the student's school context as part of the PREP intervention (*n* = *95*). An occupational therapist who was external to the study and had extensive experience working with children and youth with special needs coded the goals using a binary system according to predetermined criteria and identified the participation domains each goal addressed (see Measures section). The coding process followed an iterative consensus‐based approach. After coding the initial set of 10 goals, the researchers met to review the analysis, clarify the criteria and reach consensus on the coding. They repeated this process after an additional 50 goals were coded. Reporting follows the STROBE guidelines (von Elm et al. 2007) to ensure compliance with recognized reporting standards.

### 2.3 Measures

#### 2.3.1 Demographic Questionnaires

Therapists completed a demographic questionnaire, which included personal data, and parents completed a demographic questionnaire about their children.

#### 2.3.2 Participation‐Focused Goal Criteria

To evaluate the alignment of goals with the principles of participation‐focused practices, we developed seven criteria grounded in Cogan and Carlson's (2018) comprehensive interpretive synthesis of 76 articles on participation in the rehabilitation context. In this conceptualization, participation comprises three interrelated dimensions: (a) *performance*, the overt execution of activities in a person's life context, (b) *subjective experience*, the individual's perception and personal meaning of the activities and (c) *interpersonal connections* to others and their relations to activities. Cogan and Carlson (2018) also stress the central role of two additional concepts—choice and opportunity—in fully capturing participation.

Our criteria operationalize these dimensions in the goal analysis context:

- Performance
  1. Activity‐focused (e.g., playing in the playground, having lunch, participating in sports class).
  2. Attendance (activity frequency or duration).
  3. Real‐life settings to apply the goal (e.g., playtime, class, lunch, trips and ceremonies).
- Subjective experience
  4 Opportunity to choose. The goal reflects opportunities for the student to make choices within the activity (e.g., decide how and with whom to engage in the activity).
  5 Involvement. The goal reflects the student's active or personal engagement in the activity (e.g., affect, enjoyment and self‐initiation), rather than describing only task performance.
- Interpersonal connection
  6 Social context, including interpersonal or cultural aspects, is explicitly reflected in the goal (e.g., social relationships and culturally related activities).

Criterion 7 focuses on body functions and skills (e.g., handwriting and fine motor skills). This criterion, representing a contrasting approach to participation, emphasizes the remediation of underlying skills rather than meaningful engagement.

The seven criteria represent descriptive dimensions of participation‐focus rather than necessary or sufficient conditions for classifying a goal as participation‐focused as a whole. Each criterion was coded dichotomously, indicating the presence (1) or absence (0) of that aspect in the goal formulation. The study examined changes in each of these criteria separately across time points, and no composite index or categorical classification of goals was constructed. A detailed codebook providing operational definitions, inclusion and exclusion rules, worked examples and decision rules for borderline cases is provided as Supporting Information S1: Material A. Guided by the Occupational Therapy Practice Framework: Domain and Process (OTPF; American Occupational Therapy Association [AOTA] 2020), we also examined the participation domains that the goals addressed, allowing goals to address more than one domain. These included activities of daily living (ADL), instrumental ADL, sleep and rest, education–formal, education–informal, work, play, leisure and social participation.

### 2.4 Participants

**FIGURE 1:**
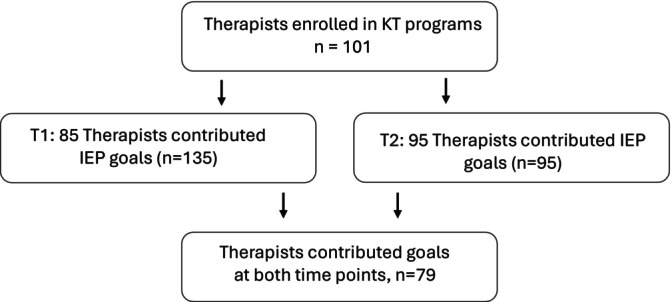
Participants' flow across study time points.

School‐based therapists (*n* = 101) included female occupational therapists (92.08%), four physiotherapists (3.96%) and four speech‐language pathologists (3.96%). The sample reflected the demographic composition of school‐based therapists employed by the Israeli Ministry of Education, with 68 participants (67.33%) from the Jewish sector, 31 (30.69%) from the Arab sector and two (1.98%) from other backgrounds. Participants had an average of 14.77 years (SD = 8.25) of professional experience; half (50.5%) held a master's degree. The children's (*n* = 101) mean age was 9.5 years (SD = 4.02, range = 3–21 years), reflecting the full age span of the Israeli special education system, which extends from pre‐compulsory kindergarten frameworks through age 21. Table 1 presents the characteristics of the children initially selected by therapists for the PREP intervention and assigned an intervention goal. Of the 101 therapists, 85 contributed one or more IEP goals at T1 (135 goals in total), 95 contributed a new goal at T2 and 79 contributed goals at both time points. Reasons for non‐contribution at a given time point were not systematically documented. Figure 1 presents the participant flow across the time points.

**TABLE 1:** Children's characteristics (*N* = 10*1*).

Characteristic	*n*	% *n*
Gender		
Boys	66	65.3
Girls	35	34.7
Educational setting		
Special education	42	41.6
Special education class in a mainstream school	15	14.9
Individual inclusion in a mainstream school	38	37.6
Other	6	5.9
Diagnosis		
Autism	32	31.7
Attention‐deficit/hyperactivity disorder	28	28.0
Cerebral palsy	23	22.8
Intellectual disability	19	18.8
Sensory impairment (hearing/vision)	8	7.9
Developmental delay	8	7.9
Mental disorder	6	5.9
Behavioural disorder	3	3.0
Other	2	1.9
≥ 2 diagnoses	35	34.6
Stakeholder involvement in goal‐formulation		
Educational professional	88	87.1
Health professional	59	58.4
Parent	68	67.3
Two stakeholders involved	43	42.5
Three stakeholders involved	36	35.6

### 2.5 Analyses

Data were analysed using SAS Version 9.4. To examine changes in goal characteristics between the two points, Generalized Linear Mixed Models (GLMMs) with a binomial distribution and a logit link function were applied. A separate model was fitted for each of the seven binary criteria, with time (T1, T2) as a fixed effect and therapist as a random intercept to account for the non‐independence of repeated observations within therapists. GLMMs are well‐suited to the unbalanced structure of the data, in which some therapists contributed goals at only one time point. For each criterion, we report the model‐estimated probability at T1 and T2, the odds ratio (OR) with 95% confidence interval for the effect of time and the corresponding *p*‐value, with *p* < 0.05 considered statistically significant. To examine the robustness of the findings to the unbalanced contribution structure, we conducted a sensitivity analysis using the same model, restricted to the 79 therapists who contributed goals at both time points. Descriptive statistics (frequencies and percentages) were used to describe the participation domains targeted by the goals across the time points. Goals could be coded under multiple domains.

## 3 Results

When comparing characteristics of IEP goals (T1) with those set during the PREP program (T2), the GLMM analyses revealed significant changes over time across all seven criteria, consistent with the expected direction following the program (Table 2). Specifically, the estimated probability of a goal being activity‐focused increased from 56% at T1 to 96% at T2 (OR = 18.50, 95% CI [6.30, 54.30], *p* < 0.001), and similar increases were observed for goals situated in real‐life settings (55% to 95%; OR = 14.57, 95% CI [5.50, 38.61], *p* < 0.001) and those addressing attendance (17% to 74%; OR = 14.25, 95% CI [7.34, 27.67], *p* < 0.001), involvement (14% to 45%; OR = 5.04, 95% CI [2.64, 9.63], *p* < 0.001), social context (54% to 91%; OR = 8.40, 95% CI [3.82, 18.48], *p* < 0.001) and choice (46% to 84%; OR = 6.03, 95% CI [3.09, 11.80], *p* < 0.001). In contrast, the probability of a goal targeting skills or body functions decreased markedly, from 38% at T1 to 5% at T2 (OR = 0.09, 95% CI [0.03, 0.23], *p* < 0.001), reflecting the expected shift from impairment‐focused to participation‐focused goals.

**TABLE 2:** Estimated probabilities and odds ratios from generalized linear mixed models comparing goal characteristics at T1 and T2.

Criterion	T1 probability	T2 probability	OR	95% CI	*p*
Activity‐focused	56	96	18.50	[6.30, 54.30]	< 0.001
Attendance	17	74	14.25	[7.34, 27.67]	< 0.001
Real‐life settings	55	95	14.57	[5.50, 38.61]	< 0.001
Involvement	14	45	5.04	[2.64, 9.63]	< 0.001
Social context	54	91	8.40	[3.82, 18.48]	< 0.001
Opportunity to choose	46	84	6.03	[3.09, 11.80]	< 0.001
Body functions/skills	38	5	0.09	[0.03, 0.23]	< 0.001

*Note:* Probabilities are model‐estimated percentages from generalized linear mixed models (binomial distribution, logit link) with therapist as a random intercept. T1 = first session (*n* = 135 goals); T2 = fifth session (*n* = 95 goals). OR = odds ratio for T2 versus T1; CI = confidence interval.

The sensitivity analysis, restricted to the 79 therapists who contributed goals at both time points, yielded a pattern of results consistent with the main analysis, supporting the robustness of the observed changes in goal formulation across the seven criteria.

Analysis of the participation domains revealed notable changes in the goals' focus. Table 3 demonstrates the goal frequency and percentage by occupational domain in T1 and T2. According to the OTPF (AOTA 2020), comparing goals at T1 with those participants set during the program (T2) revealed that formal education was the most prevalent occupational domain at both time points (T1: 71.64%, T2: 68.42%). This finding indicates that a substantial proportion of goals in both situations focused on enabling students to participate in structured, school‐based learning activities. However, compared to T1, T2 goals increased representation in play (41.05% vs. 20.3%), leisure (24.21% vs. 7.46%) and social participation (76.84% vs. 32.84%). Conversely, T1 included more goals without a coded occupational domain (14.18% vs. 2.11%).

**TABLE 3:** Goals by occupational domain: frequencies and percentages across time points.

Occupational domain	First session (T1) *N* = 135	Fifth session (T2) *N* = 95
	# (%)	# (%)
Activities of daily living (ADL)	17 (12.69)	11 (11.58)
Instrumental ADL	28 (20.90)	17 (17.89)
Sleep and rest	1 (0.75)	1 (1.05)
Education–formal	96 (71.64)	65 (68.42)
Education–informal	1 (0.75)	3 (1.05)
Work	0	0
Play	27 (20.30)	39 (41.05)
Leisure	10 (7.46)	23 (24.21)
Social participation	44 (32.84)	73 (76.84)
Uncategorised	19 (14.18)	2 (2.11)

Overall, the therapists' perceptions shifted toward goals that aligned with elements of participation‐focused practices, expanding beyond formal education into play, leisure and social participation areas. Table 4 demonstrates the goals set for a student in T1 and T2.

**TABLE 4:** Example of goal coding per participation‐focused criteria (participant 20–2023).

Criterion	First session (T1): Social play	Fifth session (T2): Play catch at recess twice a week with two friends of his choice; report enjoyment and satisfaction
Activity‐focused	1	1
Attendance	0	1
Real‐life settings	0	1
Reflects opportunities/choices	0	1
Involvement	0	1
Social/cultural context	1	1
Focus on skills/body functions	0	0

*Note:* 1 = criterion observed in the goal, 0 = criterion not observed. Goals in this example addressed the occupational domains of education–formal, play and social participation.

## 4 Discussion

Our findings suggest that a KT program based on PREP was accompanied by changes in goal formulation, with goals moving away from an impairment‐based focus toward more explicitly participation‐related dimensions. Goal‐setting stands at the heart of the IEP process in inclusive and special education school settings. It involves various stakeholders, such as therapists, teachers and parents. Thus, clear, relevant and mutually agreed‐upon goals enable focused interventions tailored to each student's needs and environmental context and provide criteria for evaluating intervention progress and outcomes (Costa et al. 2017). Participation in meaningful activities and occupations is an essential outcome in rehabilitation (WHO 2001). Therefore, participation‐focused goals are expected to be explicitly formulated rather than follow from skill‐ or body‐function‐focused interventions (Granlund and Imms 2024).

However, the absence of a universally accepted definition hinders the measurement and implementation of this concept (Cogan and Carlson 2018). Facing this challenge, Granlund and Imms's (2024) framework suggested defining participation goals as *ultimate* goals—enduring and intrinsically valuable outcomes in their own right, rather than intermediate or instrumental objectives. We propose extending their view by applying our criteria for participation‐focused goals, structured to help therapists examine whether a goal is participation‐focused and evaluate its essential dimensions (e.g., context, frequency, involvement and opportunities for choice). This, in turn, can set the stage for building participation‐focused interventions.

Nevertheless, translating evidence about participation‐based interventions into the school‐based practice context remains challenging (Zeitlin et al. 2025). Despite limited evidence that skill‐focused goals automatically lead to improved participation (Adair et al. 2015), current practice tends to emphasize skill‐ or body‐function‐oriented goals (often framed as intermediate or instrumental objectives) rather than explicitly articulating ultimate participation goals (Granlund and Imms 2024). In school settings, therapists often focus on improving skills that address body functions, such as fine motor skills for writing, rather than formulate participation‐focused goals that embed such skills within meaningful activities (e.g., completing a homework assignment; Ryan et al. 2025).

Our current study corroborates this tendency, demonstrating that only about half of the goals set within IEPs (T1), which reflect the frameworks and practices of the Israeli IEP, addressed activities, real‐life contexts or social contexts. Conversely, the goals set during the participation‐focused KT programs (T2) demonstrated a significant shift toward contextually grounded, participation‐focused goals. Similarly, our findings indicate a shift from impairment‐focused to participation‐focused goals, evidenced by fewer goals targeting improvements in body functions. This finding is consistent with a change in participants' clinical reasoning regarding goal formulation. At the same time, it is possible that the observed shift reflects, in part, therapists who were more engaged with participation‐focused practice, which should be considered when interpreting the magnitude of the change.

Importantly, clinical reasoning that incorporates goal formulation is inherently complex, reflecting the multifaceted nature of human functioning. Skills and body functions should not be disregarded. Instead, we suggest that therapists incorporate them within the complex clinical reasoning of the child's participation in meaningful activities. Indeed, Anaby et al. (2020) demonstrated that participation‐focused interventions can also lead to significant improvements in body function levels.

Our findings further highlight participation's multiple dimensions (Cogan and Carlson 2018; Imms et al. 2017), as evidenced by changes in therapists' attention to the goals' attendance and involvement dimensions, reflecting opportunities and choices. Attendance showed the most substantial difference among the criteria. Given the concrete nature of this criterion, therapists could more readily incorporate it into goal formulation. Changes were also evident in how the child's subjective involvement, experiences and available opportunities and choices were addressed. By including the child's perspective, therapists recognize the importance of what the children do as well as how they experience and take an active role in participation. This finding resonates with a child‐centred, strengths‐based approach that emphasizes children's perspectives, preferences and capacities as central to fostering meaningful and empowering participation (de Camargo 2025).

The change in participants' perspectives was evident in the participation domains that the goals addressed. Compared to the much larger proportion of goals without a coded domain reported in IEPs (14.18%), the low proportion after the program (2.11%) indicates a shift toward an occupational focus and school‐based, participation‐focused outcomes. Although formal education remained the most prominent focus at both time points, T2 goals also expanded to include play, leisure and social participation. These areas are central to child development, peer relationships and overall well‐being, yet are often overlooked in educational goal‐formulation processes (McAnuff et al. 2023; Zeitlin et al. 2025). The increased representation of these domains at T2 suggests that therapists viewed school as more than an academic setting, recognizing its role in fostering a sense of belonging, enjoyment and self‐development.

These changes in goal focus must be considered in the broader context of the present study—a structured, participation‐focused KT program, in which formulating a participation‐focused goal was integral to the curriculum (Waisman‐Nitzan et al. 2022). That study documented, using independent measures, improvements in therapists' knowledge of PREP principles, self‐perceived changes in their practice behaviours and significant gains in children's participation outcomes as measured by the Canadian Occupational Performance Measure (COPM), with changes maintained at follow‐up. Further qualitative work from the same KT initiative (Waisman‐Nitzan et al. 2022) has examined therapists' perspectives on implementing participation‐focused practice through focus groups, providing complementary insight into therapists' reasoning and the perceived mechanisms of change. Taken together with the present findings, these converging lines of evidence from the same KT initiative indicate that the changes in goal formulation observed here occurred alongside documented changes in therapist knowledge, practice behaviours and student participation outcomes. Further, the findings align with the Participation‐KT roadmap of Anaby et al. (2021), which emphasizes the potential of targeted KT initiatives to support sustainable shifts in practice. The multi‐component structure of the KT program, combining structured training, collaborative peer‐learning and ongoing coaching, is consistent with strategies commonly reported to support the uptake and sustainability of evidence‐based interventions (Flynn et al. 2023). Nevertheless, translating these changes in goal formulation into sustained changes in school‐based practice and children's real‐life participation will require multilevel, collaborative KT strategies engaging children, families, service providers, administrators and policymakers.

## 5 Limitations

Although the participation‐focused criteria were grounded in prior research and developed by experts, a broader panel did not test them, potentially limiting external validity. Goals were identified within the context of a training program, leaving unclear whether changes in therapists' practices were translated or sustained into participation‐focused intervention plans. Moreover, because the KT program combined the PREP framework and goal‐formulation practice, the present design cannot disentangle the contribution of each component to the observed changes. The coding procedure has several methodological limitations: it followed a consensus‐based approach rather than an independent parallel coding design, the primary coder was not blinded to time point and the coding scheme should be regarded as preliminary, warranting further development. The consistency of changes across all criteria and participation domains supports the robustness of the observed pattern, although the findings should still be interpreted with these limitations in mind. Finally, because most participants were occupational therapists, the findings may primarily reflect their perspectives rather than those of a broader range of school‐based rehabilitation professionals.

## 6 Conclusions

This study's exploratory findings suggest that participation in a KT program was accompanied by changes in goal formulation toward more explicitly participation‐related dimensions. The seven criteria for participation‐focused goals can serve as a useful checklist for therapists to evaluate and set participation‐focused goals. They have the potential to broaden the scope of school‐based interventions beyond formal education to also consider play, leisure and social participation. These preliminary findings point to a direction worthy of further investigation; additional research employing more formal methodological designs is needed to examine whether these changes in goal formulation translate into sustained clinical practice and improved participation outcomes for children.

## Author Contributions

**Michal Waisman Nitzan:** conceptualization, formal analysis, writing – original draft, methodology, project administration, writing – review and editing. **Yonat Ivzori:** conceptualization, formal analysis, writing – original draft, writing – review and editing. **Barkin Kose:** writing – review and editing, formal analysis. **Dana Anaby:** conceptualization, writing – review and editing, methodology.

## Funding

The authors have nothing to report.

## Ethics Statement

The Chief Scientist of the Israeli Ministry of Education (12 008, 14.10.2020) approved this study.

## Consent

Informed consent was obtained from all participants and from parents for the use of their child's de‐identified data.

## Conflicts of Interests

The authors declare no conflicts of interest.

## Supporting information


**Data S1:** Supporting Information.

## Data Availability

The data that support this study's findings are available in Hebrew and are not publicly accessible. Data may be obtained from the corresponding author upon reasonable request.
