# Supplementary material for: Moving Toward Participation‐Focused Goals for Students With Disabilities: Outcomes of a Knowledge Translation Program for School‐Based Therapists
Source: Child Care Health Dev. 2026 Jul 8;52(4):e70313. doi: 10.1111/cch.70313 (PMC13346516; doi:10.1111/cch.70313)
Supplement: Supplementary file 1 — Data S1: Supporting Information. [file CCH-52-e70313-s001.docx]

**Supplementary Material A**

*Preliminary Codebook for the Seven Participation-Focused Goal Criteria*

This preliminary codebook accompanies the analysis of participation-focused goal criteria reported in the main manuscript. For each of the seven criteria derived from Cogan and Carlson's (2018) interpretive synthesis of participation, the codebook provides an operational definition, inclusion and exclusion rules, two worked examples (one coded as criterion-present and one as criterion-absent), and an explicit decision rule for borderline cases. The criteria are coded dichotomously (1 = criterion present, 0 = criterion absent) and represent descriptive dimensions of participation-focus rather than necessary or sufficient conditions for classifying a goal as a whole. This codebook represents an initial operationalization of the Cogan and Carlson framework and warrants further development and external validation.

**Criterion 1: Activity-focused**

| **Operational definition** | The goal describes an activity that the student will perform, one that is observable in everyday life. |
| --- | --- |
| **Inclusion rule (code 1 if…)** | The goal names a concrete activity or occupation (e.g., participating in a class, playing a game, preparing food, dressing, shopping, riding a bus). |
| **Exclusion rule (code 0 if…)** | The goal targets isolated skills, components of function, or internal capacities without reference to an activity (e.g., "improve fine motor skills," "develop phonological awareness," "enhance self-efficacy"). |
| **Worked example (1)** | Example coded 1 (criterion present):  The student will help prepare spaghetti with classmates in the school kitchen. |
| **Worked example (0)** | Example coded 0 (criterion absent):  The student will improve fine motor skills. |
| **Borderline decision rule** | If a skill is named within the context of an everyday activity (e.g., "tie shoelaces during an afternoon leisure activity"), code 1- the activity context governs. |

**Criterion 2: Attendance**

| **Operational definition** | The goal specifies how often, where, or in what regular temporal pattern the activity will occur. |
| --- | --- |
| **Inclusion rule (code 1 if…)** | The goal contains a concrete frequency or temporal specification (e.g., "once a week," "three times a week," "every morning recess," "by the end of the semester"). |
| **Exclusion rule (code 0 if…)** | The goal describes the activity without specifying when, how often, or with what regularity it should occur. |
| **Worked example (1)** | Example coded 1 (criterion present):  The student will play a computer game three times a week during morning recess. |
| **Worked example (0)** | Example coded 0 (criterion absent):  The student will participate in and initiate structured and free play with peers. |
| **Borderline decision rule** | Vague temporal indications ("regularly," "consistently") without a quantifiable anchor are coded 0. A specific endpoint (by the end of the semester) counts as a temporal anchor and is coded 1. |

**Criterion 3: Real-life setting**

| **Operational definition** | The goal specifies the real-life setting in which the activity will take place. |
| --- | --- |
| **Inclusion rule (code 1 if…)** | The goal specifies a concrete setting from the student's everyday life (e.g., classroom, schoolyard, home, supermarket, kitchen, art class, family dinner). |
| **Exclusion rule (code 0 if…)** | The goal is context-free, refers only to a therapy or training session, or names only a skill without a setting. |
| **Worked example (1)** | Example coded 1 (criterion present):  The student will prepare pizza with his brother for a family dinner. |
| **Worked example (0)** | Example coded 0 (criterion absent):  The student will prepare a sandwich. |
| **Borderline decision rule** | "In school," without further specification, is coded 1 (school is a real-life setting). "In therapy sessions" alone is coded 0. When multiple settings are named, only one needs to be a real-life setting for coding 1. |

**Criterion 4: Opportunity to choose**

| **Operational definition** | The goal reflects opportunities created for the student to engage in the activity, or choices exercised within it. |
| --- | --- |
| **Inclusion rule (code 1 if…)** | The goal explicitly references the student's decision-making (selecting, choosing, deciding) or describes opportunities for participation within the context of the activity. |
| **Exclusion rule (code 0 if…)** | The goal prescribes a single fixed activity with no choice element, and no opportunity beyond the standard school routine. |
| **Worked example (1)** | Example coded 1 (criterion present):  The student will plan and lead origami sessions in art class. |
| **Worked example (0)** | Example coded 0 (criterion absent):  The student will complete class assignments on time. |
| **Borderline decision rule** | A goal that names an activity the student is offered or invited to (rather than required to perform) is coded 1. Routine task completion within standard school requirements is coded 0. |

**Criterion 5: Involvement**

| **Operational definition** | The goal refers to the student's subjective experience of the activity- engagement, enjoyment, satisfaction, motivation, or self-initiation. |
| --- | --- |
| **Inclusion rule (code 1 if…)** | The goal contains explicit reference to the student's experience or agency (e.g., "to his/her satisfaction," "with enjoyment," "will initiate," "by their own choice," "feeling capable"). |
| **Exclusion rule (code 0 if…)** | The goal describes only observable behavior or task completion without reference to the student's subjective experience. |
| **Worked example (1)** | Example coded 1 (criterion present):  The student will initiate an invitation to a friend from class and choose a joint activity with him*.* |
| **Worked example (0)** | Example coded 0 (criterion absent):  The student will play during one recess a day with two friends for 15 minutes (behavior without subjective component). |
| **Borderline decision rule** | Implicit positive framing (e.g., "successfully completes") is coded 0; only explicit references to the child's experience, satisfaction, or self-initiation count toward this criterion. |

**Criterion 6: Social/cultural context**

| **Operational definition** | The goal refers to the student's interaction with, or connection to, other people in the context of the activity. |
| --- | --- |
| **Inclusion rule (code 1 if…)** | The goal names specific social partners or social groupings (peers, classmates, family members, friends, a specific friend) in connection with the activity. |
| **Exclusion rule (code 0 if…)** | The goal describes an activity performed in isolation or with adult support, framed only as scaffolding (e.g., "with verbal mediation from the therapist") rather than as a social partner. |
| **Worked example (1)** | Example coded 1 (criterion present):  The student will play basketball with classmates to his satisfaction. |
| **Worked example (0)** | Example coded 0 (criterion absent):  The student will handwrite about half of what is required in class (solitary task). |
| **Borderline decision rule** | "With teacher mediation" or "with therapist support" alone is coded 0- the adult is a scaffolder, not a social partner. "Hosts a classmate at home" or "presents to peers in class" is coded 1. |

**Criterion 7: Body functions/skills (contrast criterion)**

| **Operational definition** | The goal targets the improvement of body functions, performance components, or isolated skills rather than participation in activities. |
| --- | --- |
| **Inclusion rule (code 1 if…)** | The goal names a body function or underlying skill as the explicit target (e.g., fine motor, gross motor, executive functions, handwriting skills, phonological awareness, motor planning, self-efficacy). |
| **Exclusion rule (code 0 if…)** | The goal targets an activity, occupation, or participation outcome, even if specific skills are implicit. |
| **Worked example (1)** | Example coded 1 (criterion present):  The student will develop graphomotor skills and writing abilities. |
| **Worked example (0)** | Example coded 0 (criterion absent):  The student will play basketball shooting with a friend in the kindergarten yard once a week. |
| **Borderline decision rule** | A goal can be coded 1 on both activity-focused (Criterion 1) and body functions (Criterion 2) when both elements are explicitly present; the criteria represent independent dimensions and are not mutually exclusive. |

*Note.* Coding was conducted by an external occupational therapist following an iterative consensus-based procedure with the research team, as described in the Methods section. Each criterion was coded as 1 (present) or 0 (absent) at the level of the individual goal; no composite "participation-focused" classification was constructed. This codebook is provided as a preliminary tool intended to support reproducibility and further methodological development of the coding scheme.
